# Patient safety in surgery: non-technical aspects of safe surgical performance

**DOI:** 10.1186/1754-9493-4-4

**Published:** 2010-03-18

**Authors:** George G Youngson, Rhona Flin

**Affiliations:** 1 Royal Aberdeen Children's Hospital, Foresterhill, Aberdeen, UK; 2 University of Aberdeen, Aberdeen, UK

## Abstract

The performance of operative surgery has an understandable focus placed on dexterity, technical precision, as well as the choice of procedure. There is less appreciation of the cognitive and social skills of the individual surgeon and the effect that these have on the surgical team and on patient outcome. This article highlights that impact and explores the contribution of non-technical skills to safe practice within the operating room.

## 

"Lack of non-technical skills can have lethal consequences for patients. However, the NHS [National Health Service] lags unacceptably behind other safety-critical industries, such as aviation, in this respect. Human Factors training must be fully integrated into undergraduate and postgraduate education."

Parliamentary Report into Patient Safety, London, July 2009

Contemporary surgical practice is, at first sight, more sophisticated, efficient and effective than ever before, and yet the incidence of adverse events during surgical care is reported at approximately 15% of cases [1] in retrospective series, and in reality is probably greater. The actual number of adverse events during operative treatment is not routinely charted, since some will have no clinical impact and others are remedied. Hence there is a complexity to the analysis in outcomes from surgery which precludes precision in a way that is often possible in other risk related situations. The concept that complexity is the "enemy of safety" in relation to medical sciences is well based. The consequences of this failure are huge and the global burden of adverse incidents occurring in the operating theatre is staggering and stands comparison with the mortality sustained in armed conflict. Such an incidence in the number of casualties of "friendly fire" would simply not be tolerated by the military forces of any country or state and yet these are the consequences of our well-intentioned but imprecisely executed actions in the operating theatre[2].

There is widespread appreciation of the physical hazards to patient and staff posed by sharps, needles, electric currents, radiation, contaminated body fluids, poor patient positioning, and other risks contained in the operating theatre environment. Whereas there is an expectation that the individual technical skills of the surgeon, anaesthetist and scrub nurse will blend in an imperceptible fashion to produce a successful surgical intervention, there has been less recognition of the risk to patients from failures in communication, leadership and decision making. Consequently, the surgical literature over the past decades is replete with knowledge of the techniques to successfully complete the intended procedure, and the training required to help surgeons acquire these elusive technical skills. And yet these hard-won technical skills are not sufficient to guarantee patient safety. Adverse events are typically seeded from deficiencies in non-technical skills (i.e. cognitive and social skills that complement technical skills and contribute to safe and efficient task performance). These are rarely the focus of surgical research and consequently do not normally feature in professional standards or training syllabi.

This situation is now changing as clinicians and social scientists begin to collaborate on systematic analyses of observed behaviours of operating room staff [3]. Many of their studies adopt an approach favoured in European aviation, where pilots are trained and individually assessed on non-technical skills that are protective for flight safety. An evidence base from aviation psychology on topics such as risk tolerance, team coordination, high speed decision making and the effects of fatigue on crew error defines the skill set to be trained. Similarly, in other high risk work settings, e.g. nuclear power plants, assuring competence in non-technical skills is a key component of licensing and revalidation[4].

Recently some more visionary medical institutions have embraced this aspect of professionalism. The Royal Australasian College of Surgeons, for example, explicitly incorporates non-technical skills in their competence and performance framework [5]. The contribution of non-technical skills (NTS) to surgical performance needs better recognition and this aspect of clinical surgery needs stronger advocacy to provide it with its correct status and importance.

Our experience of training surgeons in NTS [6] is that they readily embrace the material on cognition and find concepts such as working memory or situation awareness of immediate interest and relevance for their practice. Appreciation of the role of interpersonal skills in patient safety can be harder to convey; surgeons, like airline pilots in the first generation of Crew Resource Management training [7], do not always see why 'charm school' behaviours are relevant for technical performance. What they do not always realise is that inappropriate social behaviours, such as rudeness can diminish other team members' cognitive skills [8] and thus threaten the safe execution of a surgical procedure.

Communication is acknowledged as a key component of team performance but in surgery, it is often assumed to be a desirable and inherent attribute rather than being regarded as an important and acquired clinical skill, which may need to be applied with care [9]. Teamwork, whilst similarly valued and often judged to be developed to a high level in the operating room, may simply stem from a disparate collection of healthcare workers attempting to work together, rather than a cohesive group who explicitly recognize the objectives, the component tasks, and function with a shared mental model. As Gawande has so elegantly argued, the surgical checklist addresses both team communication and the risks of missing key tasks through the briefing mechanism [10].

Whether NTS are more important in elective or to emergency surgery is unclear. NTS have been studied more in the elective situation, but emergency surgery, by contradistinction, deals with the sickest patients with often unexpected conditions, in an environment which has availability of treatment facilities on a "first come - first served basis" rather than a triaged system within many hospitals. Emergency care is increasingly being re-organised and given greater priority than the position it previously occupied as a secondary consideration behind elective surgery. The relative inconsistency of the emergency team and "short notice" aspect to the work, places it at the forefront of risk from surgical error. The elevated mortality rates of emergency surgery are therefore attributable to a range of risks extending from the nature of the illness, to the level of experience of the attending staff; but also the time and timing of interventions, with an incomplete information base contributing to variable outcomes [11]. As yet, this is still the area most deficient from supervision of surgeons in training, time for preparation and familiarity between team members, and the greater challenge is that the awareness and importance of human factors in emergency surgery, is at an early stage of acceptance within the profession.

The new focus placed on safety in surgical practice has implications for training, education, research, and delivery of clinical care and as such, constitutes a major reason for cooperation among the responsible institutions. Training bodies, colleges and universities in Scotland over the last few years have worked together on a programme of non-technical skills research in anaesthesia, surgery and nursing, based on psychological methods used to study human performance in other safety-critical industries (e.g. civil aviation, fire fighting, prison service, oil industry, nuclear industry). Part of this work has resulted in the production of a classification of the safe behaviours relevant to surgery in a language that is finding acceptance internationally amongst surgeons - Non-Technical Surgical Skills for Surgeons - NOTSS http://www.abdn.ac.uk/iprc/notss[12]. The research flowing from collaboration between psychologists and clinicians has made a major contribution to surgeons, educators, and curriculum developers appreciating the importance, content, and teaching of patient safety, http://www.patientsafetyboard.org. It also reveals a pressing need to develop a better understanding of those social behaviours and cognitive skills that are needed by the individual members of an operating team to ensure safe and efficient surgical performance.

## Competing interests

The authors declare that they have no competing interests.

## Authors' contributions

The authors declare that both GY and RF contributed to the writing and preparation of this manuscript. Both authors read and approved the final manuscript.
